# Clinical Lectures on Diseases of the Eye—Paralysis of the Muscles of the Eye

**Published:** 1866-12

**Authors:** E. L. Holmes

**Affiliations:** Lecturer on Diseases of the Eye and Ear in Rush Medical College


*Clinical Lectures on Diseases of the Eye.* By E. L. HOLMES, M. D., Lecturer on Diseases of the Eye and Ear in Rush Medical College.

### PARALYSIS OF THE MUSCLES OF THE EYE.

GENTLEMEN,—Paralysis of the recti and oblique muscles of the eye, is a quite rare disease. Reference to tabulated lists of ophthalmic diseases will show you, I think, that only about one in four hundred patients, with abnormal conditions of the eye, is afflicted with paralysis of these nerves.

The disease, however, is of considerable importance, as not unfrequently confounded by the careless or inexperienced with strabismus; it is interesting also, as aiding us in the study of the action of the motor muscles of the eye.

Paralysis of these muscles is characterized by an inability to rotate the globe in the direction in which the muscle usually acts. In other words, the antagonistic muscle, by its tonic contraction, holds the globe (visual axis) turned in the direction opposite to the affected muscle. In paralysis of the external rectus, for instance, the pupil is turned towards the inner angle. In paralysis of the internal rectus, the globe is rotated outward. In paralysis of the inferior rectus the pupil is turned upwards, and *vice versa* in paralysis of the superior rectus. Paralysis may be total or partial. In the latter case it is termed paresis.

Paralysis of these muscles must be distinguished from a peculiar insufficiency of the muscles, especially of the internal recti, in which all the motions of the globes are normal in extent, and in which the visual axes are readily fixed upon any object. The insufficiency or weakness is principally experienced when the patient attempts to fix the eyes long at a time upon quite near objects. In this case the internal recti become fatigued, and then lose their power of holding the visual axis sufficiently convergent. Consequently one of them, the weaker, relaxes, and the visual axis turns from the object, causing a confusion of vision with more or less pain. The symptoms usually subside on looking at distant objects.

In case of paralysis of a muscle, the luscitas or fixed strabismus, as it has been sometimes called, is constant. The two principal symptoms of paralysis in general, are the relative position of the eyes, as seen by simple inspection, and the appearance of double images in the ordinary efforts of the patient to fix the eyes upon any object.

There are also two symptoms of secondary importance, which it is well to bear in mind—the manner in which the patient carries his head, and the manner in which he attempts to grasp an object, while the well eye is covered. It is scarcely necessary to dwell upon the manner of determining the position of the pupils (visual axis) in paralysis. Directing the patient to hold his head perfectly still, you place any small object in front of the patient, and then move it in any direction, to either side above or below, watching the movement of the affected eye. Any failure to follow the object with one of the eyes, can thus usually be readily detected. A critical investigation of the double images often requires much care on the part of the physician, and considerable intelligence on the part of the patient.

It will be found a convenient method in such examinations, to place the patient in front of a large tablet, held perpendicularly before the face in a darkened room. On placing a small lighted candle before the face of the patient, in a suitable position, two images of the light will be projected upon the tablet. The relative position of these images can be easily marked upon the tablet. From such examinations you will perceive that the image of the affected eye is projected in the direction of the paralyzed muscle.

If the superior rectus of one eye is paralyzed, the eye will be turned downward, but its image will be thrown above that of the well eye. *Vice versa*, if the inferior rectus of one eye is affected.

If the external rectus of one eye is paralyzed, the globe will be rotated inward, while its image will be projected outward. If either eye be now covered with the hand, it will be observed that the image upon the same side as the covered eye will disappear. On the other hand, in cases of paralysis of the internal rectus of one eye, in which the globe is rotated outward, the image will be projected inward. Remember, therefore, when either eye in paralysis is turned inward, there will be double images—in such a way, however, that the right image belongs to the right eye, and the left one to the left eye. But when either eye is turned outward, the image on the left side belongs to the right eye, and the image on the right side to the left eye. Hence, such images are called "crossed."

A careful review of what I said in a former lecture, regarding the action of the muscles, and of "corresponding portions of the two retinæ," will enable you to understand these points. The subject, however, requires considerable study to make it familiar.

The presence of double images is a symptom exceedingly annoying. Patients almost involuntarily endeavor to overcome this symptom, either by closing one eye, or by a peculiar position of the head. The muscles of the neck learn to act consensually, as it were, with the movements of the eye. The head is usually turned in the direction of the affected muscle. If, for instance, the right external rectus is paralyzed, the globe, of course, is turned to the left. The patient, in order to avoid the double image, turns the head to the right, as far as is required for the left eye, in looking at any particular object, to be turned toward the left. As soon as the left eye is turned as far to the left as the right eye is turned in consequence of the paralysis, the images coalesce.

On covering the well eye, objects seen by the affected eye, appear to be situated too far to one side. For instance, in paralysis of the external rectus of the left eye, the macula lutea, or point of most distinct vision, is turned to the left; hence, the image of an object is formed on the point of the retina to the right of the macula lutea, which point corresponds to objects situated farther to the left. In this case, therefore, the patient, in attempting to grasp the object, carries, almost invariably, his hand too far to the left of the object, and of course misses it.

This inability to measure distances accurately, together with the presence of double images, especially in attempting to walk, produces more or less giddiness and unsteadiness.

The distance between the two images, depends in a measure upon the degree of paralysis.

The attack is often very sudden, and sometimes comes on very slowly. It may inflame but one muscle, or all of them simultaneously.

The exact diagnosis, is sometimes difficult. While simple inspection is often sufficient, careful examination of the position of the double images, on looking in different directions, is sometimes absolutely necessary for determining an accurate diagnosis.

Our time will not admit of entering upon a full description of the mode of conducting such investigations for each muscle. I must refer you to what was recently said in reference to the manner in which the inferior and superior recti muscles influence the meridian of the retina, (perpendicular diameter of the cornea.) You will thus perceive, when the visual axis is directed towards the nasal side of the orbit from paralysis of the external rectus, (right eye, for instance,) that, in attempting to look outward and upward, the superior rectus must tend to rotate the eye in such a way that the meridian is inclined inward instead of being inclined outward, as it should be to remain parallel with the meridian of the other eye. As, you remember, the internal rectus of the other eye inclines the meridian inward, in looking upward and outward, the affected eye should be turned outward and upward, and its meridian inclined outward, through the action of the inferior oblique.

As the paralysis of the external rectus causes the eye to turn toward the inner angle, the inferior oblique, with the eye in this position, loses, for reason explained in a former lecture, its influence on the inclination of the meridian. The meridian of affected eye remains perpendicular. Hence, in paralysis of the external rectus, on looking upward and outward at objects, their images appear not only double, but in such a way that the upper portions of the misplaced images are inclined inward; on the other hand, for similar reasons, on looking downward and outward, their upper portions are inclined outward.

In paralysis of the internal rectus, the action of the oblique and superior and inferior recti, also have an influence in the inclination of one of the double images. The inclination is an important point in the diagnosis, in paralysis of the superior oblique.

For details in the study of this subject, I must refer you, among several important monographs, to an article by Graefe, in the first volume of the archive for ophthalmology, also to articles by Wells, in the London Ophthalmic Hospital Reports.

When all the branches of the oculomotor nerve are implicated, there is paralysis of the superior, inferior and internal recti, the inferior oblique, and of the levator of the upper lid. The globe often protrudes more or less, the pupil is dilated and the power of accommodation destroyed. In different cases, however, these last symptoms may vary in degree, or even be absent when all the external muscles are affected.

The principal causes of paralysis of the muscle of the eye, are syphilis, rheumatism, diphtheria, tumors implicating the nerves, inflammation of the base of the brain, penetrating wounds of the orbit, and exposure to sudden changes of temperature.

If the paralysis is not speedily removed, there is danger of atrophy of the affected muscle, and a permanent shortening of the antagonist, which necessarily, if fully established, produce incurable restriction in the motions of the eye.

The prognosis is grave, where the affection is caused by tumors, injuries, products of inflammation at the base of the brain, and in all neglected cases. In diphtheria, it is usually good, also in syphilis if treated early.

The treatment depends in a measure upon the cause. In syphilis and rheumatism, the treatment should be directed principally to these diseases. In diphtheria, the use of tonics is especially indicated.

Strychnia, iodide of potash and electricity, have been used with benefit. In contraction of the antagonistic muscle, tenotomy is sometimes performed with good results.

Nearly all the late text books on physiology and anatomy, are at fault in their discussions on this subject. My object in this lecture has been chiefly to serve as a guide in giving you the proper direction in your further investigation of these points in the physiology and pathology of the eye.

